# The Epidemiology of Invasive *Haemophilus influenzae* Non-Serotype B Disease in Ontario, Canada from 2004 to 2013

**DOI:** 10.1371/journal.pone.0142179

**Published:** 2015-11-16

**Authors:** Shalini Desai, Frances B. Jamieson, Samir N. Patel, Chi Yon Seo, Vica Dang, Jill Fediurek, Debeka Navaranjan, Shelley L. Deeks

**Affiliations:** 1 Immunization and Vaccine Preventable Diseases Division, Public Health Ontario, Toronto, ON, Canada; 2 Public Health Ontario Laboratories, Public Health Ontario, Toronto, ON, Canada; 3 Department of Laboratory Medicine and Pathobiology, University of Toronto, Toronto, ON, Canada; 4 Dalla Lana School of Public Health, University of Toronto, Toronto, ON, Canada; The Hospital for Sick Children and The University of Toronto, CANADA

## Abstract

**Background:**

Since the widespread use of *Haemophilus influenzae* (Hi) type b (Hib) vaccines among children aged <5 years, an increase in invasive non-Hib disease incidence has been reported internationally. We sought to describe the epidemiology of invasive non-Hib disease in Ontario, Canada (population ~13.5 million).

**Methods:**

Confirmed invasive non-Hib cases (non-typeable [NTHi] and serotypes a, c, d, e, and f) were obtained from the provincial laboratory data system from 2004–2013. Data were deterministically linked to the provincial reportable disease system to provide further case information. Antibiotic resistance data were analysed separately from 2010–2014. Descriptive analyses included incidence rates, age group, serotype, site of specimen collection and resistance patterns; ethnicity data were not available. Temporal trends were evaluated by Poisson regression and p-values <0.05 were considered significant.

**Results:**

A total of 1307 cases of invasive non-Hib disease were included, increasing from 0.67 cases to 1.60 cases /100,000 from 2004 to 2013. Significant increases in the incidence of NTHi (0.50 to 1.28 cases/100 000 population), Hia (0.02 to 0.08 cases/100, 000) and Hif (0.13 to 0.18 cases/100, 000 population) were seen. Among persons aged 40–64 years, 3 Hi strains significantly increased over time; NTHi (0.22 to 0.99 cases/100, 000), Hia (0.00 to 0.06 cases/100, 000) and Hif (0.05 to 0.21 cases/100, 000). Among persons aged 65–84 years, there was a significant increase of NTHi (1.62 to 3.14 cases/100, 000) and Hia (0.00 to 0.34 cases/100, 000). Among persons aged 85+ years, only NTHi significantly increased from 4.89 to 10.28 cases/100, 000). Antimicrobial resistance (AMR) to ampicillin and clarithromycin was seen in greater than 25% of isolates but AMR did not increase over the duration of this study.

**Conclusions:**

The incidence of invasive non-Hib disease has increased over time; NTHi, Hif and Hia are emerging pathogens, and should be monitored.

## Introduction

*Haemophilus influenzae* (Hi) is a Gram negative facultative anaerobic coccobacillus. Strains may be unencapsulated (non-typeable or NTHi) or have a polysaccharide capsule (serotype a-f). The organism can cause disease in humans that ranges from severe invasive infections such as meningitis and epiglottis to non-invasive infections such as acute otitis media. Prior to the introduction of *Haemophilus influenzae* type b (Hib) vaccine in the 1980’s (polysaccharide vaccine in 1986[1], conjugate vaccine in 1988[2], Hib was a common cause of invasive infections in young children[3]. In 1987, the reported incidence of invasive Hib infections in Canada was 2.53 per 100,000 population[4]. Since the introduction of Hib vaccine for children under the age of 5 years, there has been a precipitous decline in the incidence of Hib disease both in Canada[4] and in other countries with Hib immunization programs[5,6]. These trends are similar for the province of Ontario, Canada (population 13.5 million in 2014)[7]. Only 6 confirmed cases of invasive Hib were reported in Ontario, for an incidence of 0.4 cases per 1 million population in 2013[8].

With such a low incidence of invasive Hib, reports have been emerging that disease due to non-Hib strains are increasing[2,9,10]. The clinical syndromes associated with non-Hib cases have been reported to be similar, but not as severe as those related to Hib. Additionally, cases of non-Hib disease have been reported to exhibit antimicrobial resistance (AMR) to commonly used therapeutic agents[11–14] making treatment more challenging. The purpose of our study was to describe the epidemiology and AMR of invasive non-Hib infections in the province of Ontario, Canada.

## Methods

Invasive Hib is a reportable disease in Ontario. While non-Hib cases are not reportable, they are often captured in the reportable disease system prior to serotype confirmation among cases under investigation for Hib. Laboratory testing including serotype determination is conducted at the Public Health Ontario Laboratories, the provincial reference laboratory. The majority of serotyping of Hi cases occurring in Ontario (>95%) is performed at Public Health Ontario Laboratory.

We included confirmed invasive non-Hib cases (nontypeable [NTHi] and serotypes a, c, d, e, and f) from January 1, 2004 to December 31, 2013 obtained from the Public Health Ontario Laboratory information system in the analysis. Variables contained in the data include serotype, source of specimen collection, specimen received date, patient’s name, age/date of birth, sex, and region of residence (postal code). Only isolates from sterile sites such as cerebrospinal fluid (CSF), blood, joint aspirates, thoracentesis fluid, and tissue biopsy specimens, were included. Excluded from the analysis were Hib cases, non-Hi isolates (e.g. *H*. *parainfluenzae*), isolates from individuals who reside outside of Ontario, isolates without patient name, and isolates without any serotype information (i.e. typing data was absent from either database). Some individuals were associated with multiple isolates, sometimes collected from multiple sites. If the isolates from the same individual were received within five months of each other, they were considered to be part of the same episode. Only one case per episode was included in the analysis, however multiple specimens could be included with a case.

Where available, we linked data from Public Health Ontario Laboratory to data extracted from the provincial reportable disease system (the integrated Public Health Information System) based on patient’s name, gender, age and/or date of birth, and date of onset of infection for the study period (2004–2013). This linkage was done to validate patient demographic information collected from the laboratory requisition forms and to retrieve missing demographic information. If there was discordance within a linked file, demographic data from the integrated Public Health Information System was considered the source of truth while serotyping information from the lab was considered the source of truth. We extracted data from the integrated Public Health Information System on November 18, 2014. Ethnicity data were not available in either data set.

Conventional biochemical testing was used to identify and confirm *H*. *influenzae* species. The invasive isolates were serotyped by slide agglutination method using commercial anti-sera to capsular antigens a-f (Remel Europe Ltd, Dartford, England)[15]. In addition, antimicrobial susceptibility testing (AST) was performed using broth microdilution method, (Thermo Fisher Scientific, Oakwood Village, OH), and minimum inhibition concentration (MIC) results were interpreted based on Clinical and Laboratory Standards Institute[16]. Antimicrobial testing data was only available from 2010–2014 and these data were not linked to the integrated Public Health Information System data.

We calculated incidence per 100,000 population using annual population denominators from Statistics Canada, (2004–2013), obtained through intelliHEALTH Ontario and the Ontario Ministry of Health and Long-Term Care. Public health unit information from iPHIS and laboratory records was used to group cases into seven health regions for geographic analysis and mapping. For those isolates with public health unit information obtained from the laboratory data, only those with a patient postal code were included in the analysis to exclude those cases that use public health unit of the health-care provider as a proxy for patient public health unit (n = 120). We evaluated temporal trends in incidence using Poisson regression and differences in proportions using Fisher’s exact test. P-values <0.05 were considered significant. We conducted analyses using SAS version 9.3 and Microsoft Excel 2010.

We obtained ethics approval from Public Health Ontario’s ethics review board. All patient information was anonymized and de-identified prior to analysis.

## Results

### Overall

**Fig 1:**
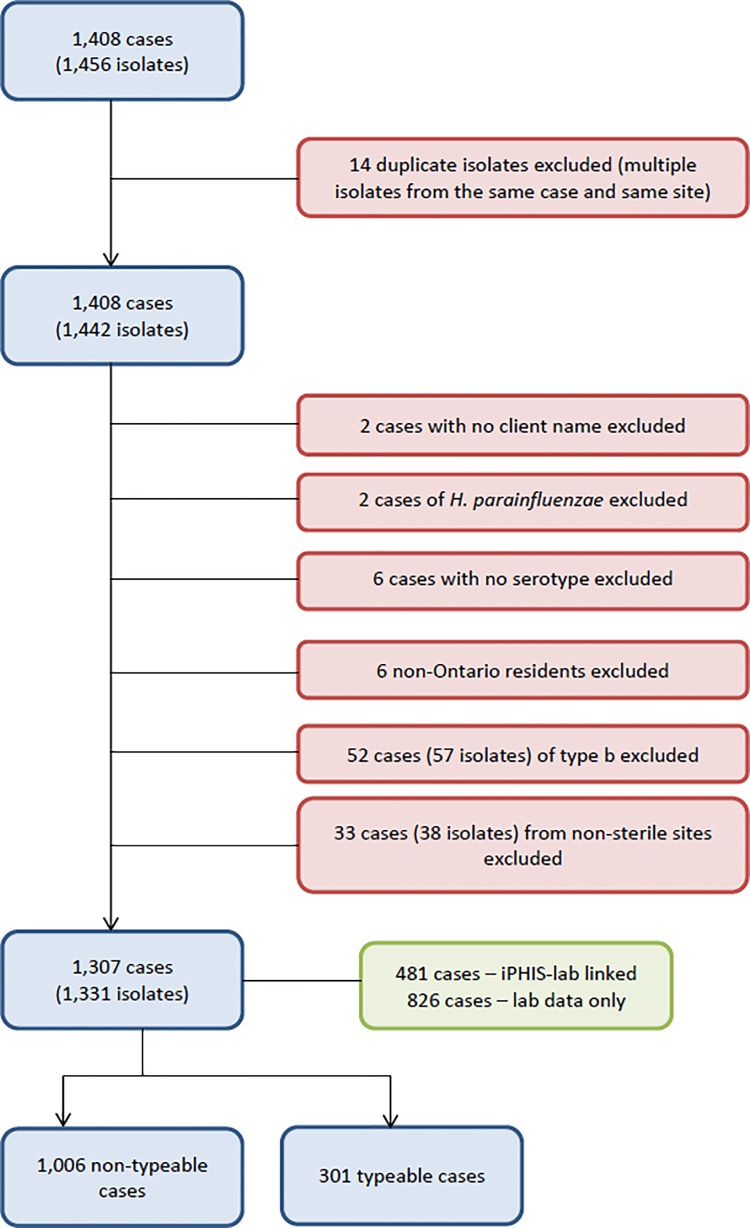
Data abstraction, cleaning and linkage for non-Hib cases 2004–2013, Ontario, Canada.

A total of 1,456 isolates, representing 1,408 distinct cases of invasive non-Hib disease were reported from the Public Health Ontario Laboratory data between 2004 and 2013 in the province of Ontario. (Fig 1) A total of 1,307 cases (1331 isolates) met inclusion criteria. Only 481 cases (36.8%) were linked to the integrated Public Health Information System data for validation and additional retrieval of demographic information.

**Fig 2:**
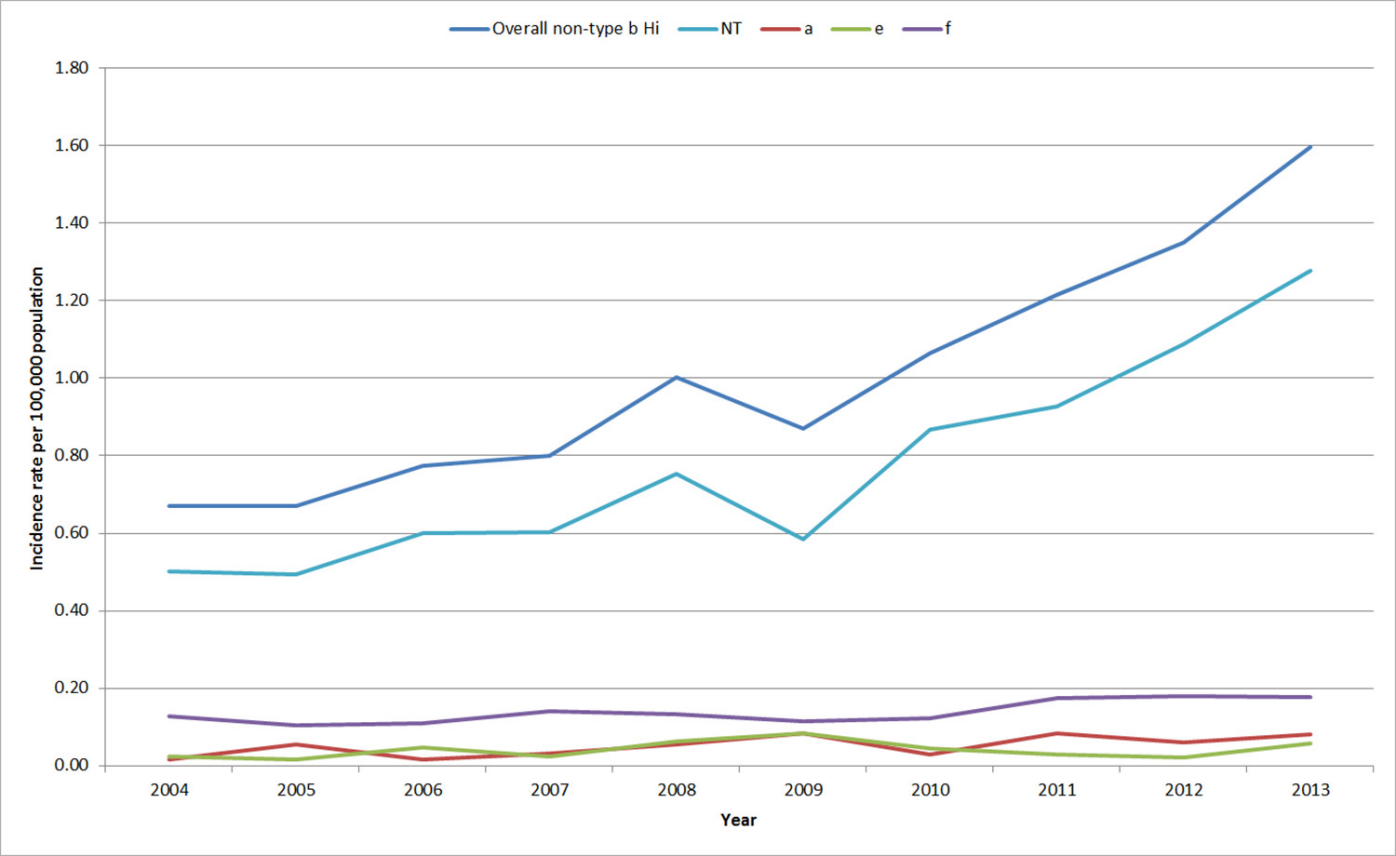
Incidence rate of invasive non-type b Hi by serotype in Ontario, 2004–2013 (n = 1,307).

There were 301 typeable cases (23%) and 1,006 NTHi cases. Of the 301 typeable cases, 67 (22.3%) were serotype a, 54 (17.9%) were serotype e, 180 (59.8%) were serotype f and there were no serotype c or d isolates. The overall incidence rates of non-Hib infections significantly increased over the study period (p<0.05) from 0.67 cases in 2004 to 1.60 cases per 100,000 population in 2013. The majority of this increase in incidence was due to NTHi. The proportion of total cases that were NTHi varied from 67.3% in 2009 to 81.4% in 2010. During the period under surveillance there was a significant increase in the incidence of NTHi (0.50 to 1.28 cases/100,000 population), as well as Hia (0.02 to 0.08 cases/100,000) and Hif (0.13 to 0.18 cases/100,000 population) (p<0.05). (Fig 2)

### Age

**Fig 3:**
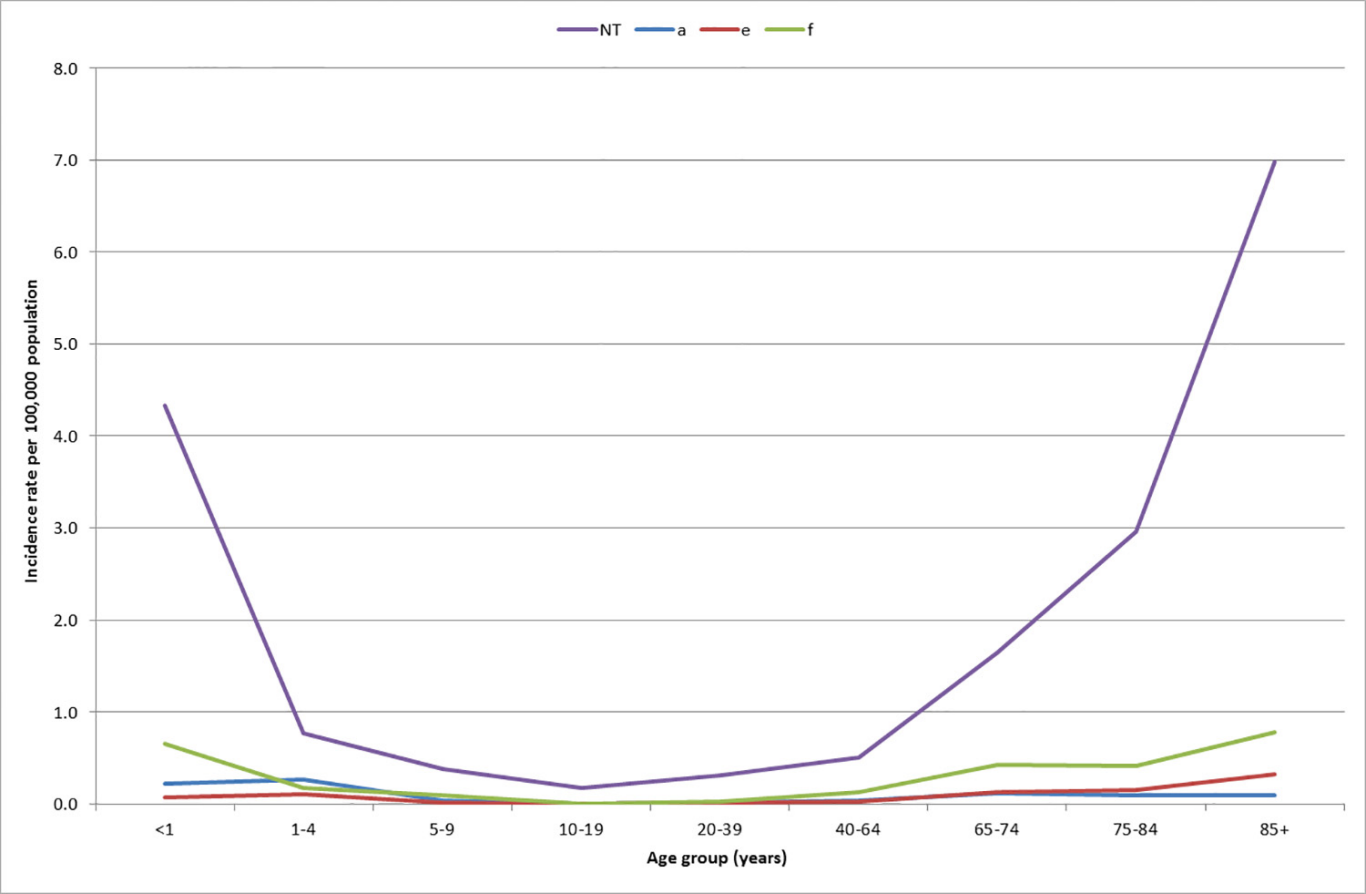
Incidence rate of invasive non-type b Hi by serotype and age group in Ontario, 2004–2013 (n = 1,290).

**Fig 4:**
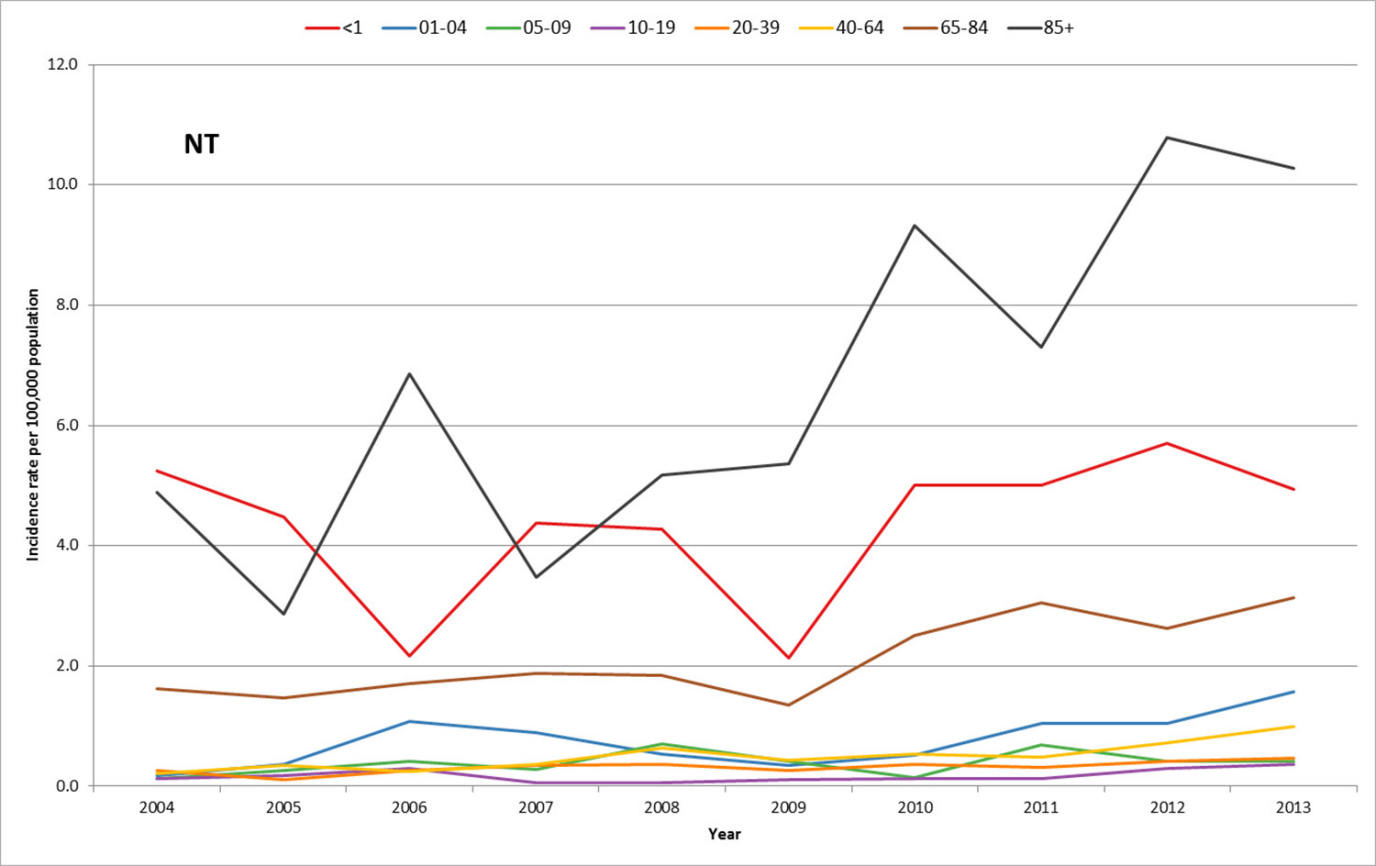
Incidence rate of invasive non-typeable Hi by age group in Ontario, 2004–2013 (n = 990).

Information on age was available for 1,290 (98.7%) cases. Cases ranged in age from younger than 1 month to 102 years. Children under the age of 1 year and individuals 85 years of age and older had the highest incidence rate, creating a ‘U’ shaped curve. The highest incidence of non-Hib disease was observed in adults 85 years of age and older (8.17 cases per 100,000 population) with NTHi accounting for 85.4% of cases (6.98 cases per 100,000 population). Children under the age of 1 year of age had the second highest incidence of non-Hib disease (5.27 cases per 100 000 population), again with the 82.2% of cases due to NTHi isolates (4.33 per 100,000 population). When all of those 65 years of age and older were analyzed together, their rate of NTHi was 2.75 per 100,000 population. (Fig 3) Among adults 20 years of age and older, there was a statistically significant increase in the overall non Hib incidence of infection. For those 40–64 years of age, three Hi strains significantly increased over time; NTHi (0.22 to 0.99 cases/100,000 population), Hia (0.00 to 0.06 cases/100,000 population) and Hif (0.05 to 0.21 cases/100,000 population). Among persons 65–84 years of age, there was also a significant increase of NTHi (1.62 to 3.14 cases/100,000 population) and Hia (0.00 to 0.34 cases/100,000 population). However, among persons 85 years of age and older only NTHi significantly increased from 4.89 to 10.28 cases/100,000 population). (Fig 4)

### Geography

There were 778 (59.5%) cases with either health region entered into the integrated Public Health Information System and /or patient postal code information entered into the laboratory data. Mapping of cases and incidence of disease by health region showed no geographical variation in the incidence of infection or the specific serotype, including in the northern regions of the province (data not shown).

### Specimen types

Of the 1,331 non-Hib isolates from 1,307 distinct cases, 91.7% (1220/1331) were from blood, 4.8% (64/1331) were from CSF and the remaining isolates were from other sources including joint fluid, biopsy specimens, empyema/pleural fluid, abscesses and vitreous fluid. The number of isolates was greater than the number of cases because a case could have more than one isolate cultured from more than one site. There was no significant association between specimen types and isolate serotype, 91.8% (934/1017) versus 4.4% (45/1017) of NTHi were from blood and CSF respectively, while 91.1% (286/314) versus 6.1% (19/314) of typeable non-Hib were from blood and CSF.

### Antimicrobial susceptibility testing

AST information was available for all non-Hib isolates received by Public Health Ontario Laboratory from January 2010-December 2014 (n = 664). Similar to the larger dataset presented above, the majority of isolates with AST data available were recovered from blood (n = 584; 87.9%), followed by CSF (n = 30; 4.5%). Thirty one (4.7%) non-Hib isolates were serotyped as a, 19 (2.9%) e, 84 (12.7%) f, and 530 (79.8%) isolates were NTHi.

NTHi isolates (n = 530) were found to have increased antimicrobial resistance compared to non-Hib typeable (a, e, f) isolates (n = 134) (p<0.001). Specifically, 30% of NTHi isolates were resistant to clarithromycin, while 14.2% of typeable non-Hib isolates were resistant (p<0.001). 26.4% of NTHi isolates were resistant to ampicillin as compared to 6.7% of typeable non-Hib strains (p<0.001). Resistance against ampicillin/sulbactam and cefaclor was also more common among strains of NTHi. There was no resistance documented in any of the non-Hib strains to third generation cephalosporins or to meropenem (Table 1).

**Table 1:** Antimicrobial resistance of invasive non Hib strains isolated in Ontario, 2010–2014 (n = 664).

	a,e,f serotype					Non-typeable				
Antibiotic	N	MIC_50_	MIC_90_	Range	%NS^#^	N	MIC_50_	MIC_90_	Range	%NS^#^
Amoxicillin-clavulanate	134	≤2	≤2	≤2–4	2.2	529	≤2	≤2	≤2–8	2.6
Ampicillin	134	≤0.12	1	≤0.012 -- >4	6.7	530	0.25	1	≤0.12–4	26.4
Ampicillin-sulbactam	134	≤1	≤1	≤1 -- >2	2.2	529	≤1	2	≤1–2	7.5
Cefaclor	134	≤4	8	≤4 -- >16	3.7	524	≤4	8	≤4–8	11.4
Ceftriaxone	134	≤0.03	0.06	≤0.03–1	0	530	≤0.03	≤0.03	≤0.03–0.25	0
Clarithromycin	134	8	16	≤0.12–16	14.2	530	8	8	≤0.12 -- >16	30
Levofloxacin	134	≤0.03	≤0.03	≤0.03–1	0	530	≤0.03	≤0.03	≤0.03 -- >4	0.2*
Meropenem	134	≤0.06	≤0.06	≤0.06–0.5	0	530	≤0.06	0.12	≤0.06–0.5	0

*indicates resistance breakpoints have not been established

#indicates both resistance and intermediate category. Non-susceptible and intermediate (= NS)

## Discussion

Internationally, the Global Alliance for Vaccines and Immunization (GAVI) has been funding Hib vaccine since 2005[17]. As of 2013[18], all GAVI eligible countries have introduced the vaccine. Other jurisdictions have published their experience with non-Hib infections and, similar to our findings have also documented a gradual increase in the incidence of invasive non-Hib infections[9,19,20].

The largest increase in incidence in our study was due to NTHi in infants and older adults. We also observed a smaller increase in the incidence of Hia overall. Ladhani and colleagues in their study reviewing the epidemiology of invasive Hi disease in Europe from 1996 to 2006, included a subset analysis of years 2000–2006 when vaccine had been introduced in all fourteen study countries. They found the highest incidence of disease among those younger than 1 year of age and those 65 years of age and older. In this study, as in our study, infants had a higher incidence (2.1 cases per 100 000) than adults65 years of age and older (0.7 cases per 100 000)[19]. However, we observed higher incidence rates in both age groups (5.27 per 100 000 and 2.75 per 100 000 respectively). This difference in incidence may have been due to an inadequate amount of time lapse between introduction of the vaccine and review of surveillance data. In Ontario, vaccine was introduced in 1998 and our study period began in 2005. In the Ladhani study, some countries would have introduced vaccine in 2000, at the same time that they were reporting incidence of Hi. In Queensland, Australia Cheong and colleagues reported on the incidence of Hi from 2000 to 2013[20]. NTHi was observed across the age spectrum with the highest rates seen in adults over 60 years (incidence of 7.0 cases per 100,000 population). Over their study period, they also showed a significant increase in NTHi, again with infants and the elderly carrying the largest burden of disease. In the US[9], as a part of their Active Bacterial Core surveillance (ABC), they also found that the incidence of NTHi cases was the highest in those younger than 1 year of age (5.87 cases per 100,000) and those 65 years of age and older (4.09 cases per 100,000). The Canadian province of Manitoba showed an increasing proportion of invasive cases due to non-Hib with a wide distribution across different ages[21]. The variation in rates observed in these studies could be due to a number of factors including location specific testing patterns, variation in recognition of Hi as a causative organism in clinical practice that leads to more testing of older adults or prevalence of risk factors within a specific study population. Together these data do suggest that NTHi cause a substantial burden of invasive infections in young infants and in older adults.

Most isolates of NTHi in our study were from the blood and CSF; only 2% were from empyema or pleural fluid, with over 95% of these NTHi. This is consistent with other studies[9] in which blood and CSF are the majority of specimen types. We could not classify cases based on clinical syndrome due to a lack of associated clinical information. In the literature, pneumonia is the most frequent clinical syndrome[9,20] especially in older adults. Cases of pneumonia would not have met our case definition unless there was an isolate from a normally sterile site (i.e. bronchoalveolar lavage, empyema or pleural fluid). The Infectious Diseases Society of America (IDSA) recommends choosing antibiotics with *H*. *influenzae* coverage in adults with community acquired pneumonia[22]. Other studies have suggested that NTHi is an opportunistic organism in those than 65 years of age and older, who have immunosuppressing or chronic respiratory disorders[23–25]. Further data on case risk factors would be helpful in understanding the epidemiology we observed in our study.

Hi typeable serotypes were also found to contribute to disease burden in Ontario. The most significant increase was seen in Hif and Hia. Although one study did not report a significant increase in Hi typeable serotypes [20], an increase in incidence has been reported by others. Hia[26–28] has been increasing in incidence in Northern locations, especially among aboriginal populations. In a population based study of the Canadian Circumpolar region, the incidence of Hia was reported as 4.6 cases per 100,000 population over the period of 2000 to 2010. Over 90% of these cases were in individuals of Aboriginal heritage. The site of infection was similar to that seen with NTHi strains, the majority of isolates were from blood and CSF.

Our data did not contain information on aboriginal status, but one would expect that in the Northern region of the province, where about 30% of the population is of Aboriginal heritage[29], there would have been an increase in rates of non-Hib serotypes. Brown and colleagues, specifically reported on a high incidence of non-Hib cases in the northern region of Ontario. In 2007, the overall incidence among all age groups was reported as 2.98 cases per 100, 000 population. In this study, 52.6% (20/38) were of Aboriginal heritage. We did not see higher rates of disease in the norther region of the province in our analysis. This may have been due to exclusion of records without postal code listed in our dataset or instability of our rates due to small population size in the North. Alternatively, this could be due to the small sample size in the previous study associated with unstable rates.

We found greater than 10% resistance to cefaclor (11.4%), ampicillin (26.4%) and clarithromycin (30%) among NTHi cases. These results suggest ampicillin and clarithromycin as empiric therapy should not be used in the treatment of NTHi infections, since treatment failure as a result of resistance could occur. Other studies have shown variable rates of AMR in NTHi isolates. In a small study of children with acute otitis media, middle ear fluid as well as NP swabs taken at the time of infection showed 16.7 to 56.2% beta lactamase positive NTHi strains over a four year period[11]. In a review by Van Eldere and colleagues, beta lactamase resistance among NTHi isolates was found to be between 5–25%[30]. This variation in AMR seen in different jurisdictions is likely due to differential antimicrobial use. When comparing the resistance between NTHi and typeable Hi, there was a significant difference overall with NTHi showing more resistance than typeable Hi. Resistance to commonly used first line antimicrobials for meningitis[31], and sepsis[32] such as ceftriaxone and meropenem was not found. Over the 4 year period that was included in this study, no increase in AMR was noted among commonly used antibiotics. In the literature, there have been some reports of increasing AMR[30,33] specifically among NTHi strains over time. Based on the known effect of antibiotic usage pressure, further studies would need to be done to see if AMR increases over time within our population.

In Ontario, non-Hib infections are not reportable, therefore it was not surprising to have only 36.8% of our cases reported in both the integrated Public Health Information System and in the laboratory data. As there has been an observable increase in incidence in non-Hib invasive infections, particularly from blood and CSF adding non-Hib to the list of diseases under provincial surveillance would facilitate further monitoring of non-Hib trends. This would allow for monitoring of disease incidence, further characterization of those at highest risk, provide valuable AMR information to guide empiric antibiotic choices and provide additional information for possible vaccine development[34].

### Limitations

As with any observational study, there are limitations to our study. Isolates may not have been submitted for serotyping at Public Health Ontario Laboratory and some cases may have been missed if specimens were not collected for culture in a timely fashion (i.e. before starting antibiotics). Alternatively, it is possible that more testing is being done and our analysis did not adjust for the number of samples submitted. No data were available on risk factors (underlying medical conditions, ethnicity) and no data were available on outcomes. The lack of data on ethnicity is of particular relevance since aboriginal populations have been found to have a higher incidence of infection with Hi[9,20,26,27]. The absence of postal code data for 40.7% patients is another limitation which may have resulted in an underestimate of incidence by health region, especially if data were more likely to be missing from specific regions of the province. Further AMR testing was not available to determine specific types of resistance present within the isolates included.

## Conclusions

The incidence of invasive non-Hib disease has increased over time in Ontario, Canada and our data suggest that NTHi, Hif and Hia are emerging pathogens which should be monitored. Non-Hib infections are causing serious invasive infections especially in the most vulnerable individuals at the extremes of the age spectrum (i.e., those under the age of 1 year and those 65 years of age and older). Adding all invasive Hi, to the reportable diseases list for Ontario would improve data quality and allow for additional risk factors to be characterized, allow improved monitoring of trends and guide appropriate empiric antibiotic choices within our population.
